# Draft Genome Sequence of Carbapenemase-Producing *Serratia marcescens* Isolated from a Patient with Chronic Obstructive Pulmonary Disease

**DOI:** 10.1128/genomeA.01288-17

**Published:** 2017-11-16

**Authors:** Sarah Lepuschitz, Sieglinde Sorschag, Burkhard Springer, Franz Allerberger, Werner Ruppitsch

**Affiliations:** a Institute of Medical Microbiology and Hygiene, Austrian Agency for Health and Food Safety, Graz, Austria; b Vienna University of Technology, Research Area of Biochemical Technology, Institute of Chemical, Biological and Environmental Engineering, Vienna, Austria; c Department of Hospital Hygiene and Infectious Diseases, Community-Hospital Klagenfurt am Wörthersee, Klagenfurt, Austria; d Department of Biotechnology, University of Natural Resources and Life Sciences, Vienna, Austria

## Abstract

The occurrence of multidrug-resistant *Serratia marcescens* strains producing metallo-β-lactamases or extended-spectrum β-lactamases represents a serious public health threat. Here, we report the draft genome sequence of a multidrug-resistant carbapenemase-producing *Serratia marcescens* isolate recovered from the bronchoalveolar lavage specimen of a patient suffering from chronic obstructive pulmonary disease (COPD).

## GENOME ANNOUNCEMENT

*Serratia marcescens*, first described in 1819, belongs to the family of *Enterobacteriaceae* and is a motile rod-shaped Gram-negative bacterium (1). *Serratia* species are omnipresent in the environment, and *S. marcescens* is classified as an important nosocomial pathogen causing a wide range of infections, including, most notably, urinary tract infection and bloodstream infection (2). Besides several potential virulence factors, one important feature of clinical *S. marcescens* is its ability to acquire antimicrobial resistance. VIM-metallo-β-lactamase (MBL)-producing isolates have the ability to hydrolyze almost all β-lactams and have been described in association with outbreaks worldwide (3).

In 2017, in Austria, *S. marcescens* strain at10508 was cultured from a 68-year-old male patient with clinical signs of chronic obstructive pulmonary disease (COPD), pneumonia, brain abscess due to a *Nocardia* sp., diabetes mellitus type 2, liver cirrhosis, coronary heart disease, ascites, and pleural effusion. Antimicrobial resistance was determined using BD Phoenix (Becton Dickinson, Franklin Lakes, NJ, USA), yielding the following results: ampicillin (resistant [R]), ampicillin-sulbactam (R), amoxicillin-clavulanic acid (R), piperacillin (R), piperacillin-tazobactam (R), cefazolin (R), cefepime (R), cefotaxime (R), ceftazidime (R), cefuroxime (R), ertapenem (R), imipenem (R), meropenem (R), ciprofloxacin (R), levofloxacin (R), amikacin (sensitive [S]), gentamicin (S), tobramycin (R), tetracycline (R), tigecycline (intermediate [I]), colistin (R), fosfomycin (R), and trimethoprim-sulfamethoxazole (R).

For whole-genome sequencing, high-molecular-weight DNA was isolated from an overnight culture on Mueller Hinton agar plates (BioMérieux, Marcy-l'Étoile, France) using the MagAttract HMW DNA kit (Qiagen, Hilden, Germany). Using the double-stranded DNA (dsDNA) BR assay kit (Thermo Fisher Scientific, Waltham, MA, USA), 1 ng of input DNA was quantified with a Qubit 2.0 fluorometer (Thermo Fisher Scientific). Library preparation to obtain ready-to-sequence libraries was done with a NexteraXT kit (Illumina, Inc., San Diego, CA, USA). Paired-end sequencing (2 × 300 bp) was performed using a MiSeq system (Illumina, Inc.) and generated 3,174,214 reads from 687,587,445 unassembled nucleotides. Raw reads were *de novo* assembled into a draft genome using SPAdes version 3.9.0 (4). Contigs were filtered for a minimum coverage of 5 and minimum length of 200 bp, which resulted in 272 contigs with a total of 5,687,772 nucleotides at a coverage of 133-fold.

Antimicrobial resistance genes were identified using the ResFinder tool (5) from the Center of Genomic Epidemiology (CGE) (http://www.genomicepidemiology.org) and included *bla*_VIM-1_, *bla*_ACC-1_, *bla*_SRT-2_, *aadA1*, *aadA16*, *aac(6')-Ic*, *aac(6')Ib-cr*, *qnrB6*, *tet*(41), *dfrA27*, *arr-3*, *catA1*, and *sul1*, conferring resistance to β-lactam antibiotics, aminoglycosides, quinolones, tetracyclines, trimethoprim, rifampin, phenicol, and sulfonamides. The PlasmidFinder tool from CGE (6) identified two plasmids (IncHI2, IncHI2A). The NCBI Prokaryotic Genome Automatic Annotation Pipeline identified 5,674 genes, 5,552 coding sequences, 194 pseudo-genes, 19 rRNA operons (9 complete, 10 partial), and 82 tRNA genes.

### Accession number(s).

This whole-genome shotgun project has been deposited at DDBJ/ENA/GenBank under the accession number NPIX00000000. The version described in this paper is version NPIX01000000.
